# Combined IL-12 Plasmid and Recombinant SjGST Enhance the Protective and Anti-pathology Effect of SjGST DNA Vaccine Against *Schistosoma japonicum*

**DOI:** 10.1371/journal.pntd.0004459

**Published:** 2016-02-18

**Authors:** Po-Ching Cheng, Ching-Nan Lin, Shih-Yi Peng, Tsung-Fu Kang, Kin-Mu Lee

**Affiliations:** 1 Department of Molecular Parasitology and Tropical Diseases, School of Medicine, Taipei Medical University, Taipei, Taiwan; 2 Institute of Microbiology and Immunology, National Yang-Mng University, Taipei, Taiwan; 3 Department of Biochemistry, College of Medicine, Tzu Chi University, Hualien, Taiwan; 4 Institute of Tropical Medicine, National Yang-Mng University, Taipei, Taiwan; 5 Institute of Biotechnology, Central Taiwan University of Science and Technology, Taichung, Taiwan; Universidade Federal de Minas Gerais, BRAZIL

## Abstract

Schistosomiasis is listed as one of most important tropical diseases and more than 200 million people are estimated to be infected. Development of a vaccine is thought to be the most effective way to control this disease. Recombinant 26-kDa glutathione S-transferase (rSjGST) has previously been reported to achieve a worm reduction rate of 42–44%. To improve the efficiency of the vaccine against *Schistosoma japonicum*, we immunized mice with a combination of pcDNA vector-encoded 26-kDa SjGST (pcDNA/SjGST), IL-12 expressing-plasmid (pIL-12), and rSjGST. Co-vaccination with pcDNA/SjGST, pIL-12, and rSjGST led to a reduction in worm burden, hepatic egg burden, and the size of liver tissue granulomas than that in the untreated infection controls. In addition, we detected high levels of specific IgG, IgG1, and IgG2a against the rSjGST antigen in infected mice vaccinated with this combination of pcDNA/SjGST, pIL-12, and rSjGST. Moreover, high expression levels of Th2 cytokines, including IL-4 and IL-10, were also detected in this group, without diminished levels of IL-12, INF-γ, and TNF-α cytokines that are related to parasite killing. In conclusion, we have developed a new vaccination regimen against *S*. *japonicum* infection and shown that co-immunization with pcDNA/SjGST vaccine, pIL-12, and rSjGST has significant anti-parasite, anti-hepatic egg and anti-pathology effects in mice. The efficacy of this vaccination method should be further validated in large animals such as water buffalo. This method may help to reduce the transmission of zoonotic schistosomiasis japonica.

## Author Summary

Schistosomiasis continues to be a serious global public health problem that considered by World Health Organization (WHO). More than 200 million people are infected and cause 280 thousand deaths every year. Among, *Schistosoma japonicum* causes the most severe pathological damages and the slowest immune resistance manifestation. It is estimated there is considerable economic loss in China due to the infection of human and domestic animals. Therefore, development of a useful vaccine is thought to be an efficient strategy to control and prevent schistosome infection. In this study, we co-immunized mice with pcDNA/SjGST vaccine, pIL-12 and rSjGST to develop a new vaccination regimen against schistosomiasis japonica. And we found this regimen can induce both specific cellular and humoral responses to attain a balance between parasite elimination and prevention of pathological tissue injury. The new regimen produced significant anti-parasite, anti-hepatic egg, and anti-pathology effects. Our method of vaccination can be applied in large livestock, such as water buffalo or cow that may help to reduce the transmission of zoonotic schistosomiasis japonica.

## Introduction

Schistosomiasis is an important helminth parasitic disease, and it remains a major health problem worldwide, especially in tropical and subtropical countries [1]. *Schistosoma japonicum* causes the most severe pathological symptoms, and it is estimated that several million people in China are infected every year, with considerable economic loss due to infection of both humans and domestic animals [2, 3]. Although effective chemotherapeutic drugs, such as praziquantel and artemether (artemisinin derivatives), are available for the treatment and prevention of schistosomiasis [4], reinfection and decreased susceptibility to the drugs restrict their effectiveness [5]. Therefore, development of a safe and efficient vaccine would be a better strategy for control and prevention of schistosome infection [6].

Progress continues in the development of an anti-schistosomiasis vaccine. Sjc26GST (*S*. *japonicum* 26-kDa glutathione *S*-transferase; GenBank accession no. BU711548.1) is a potential vaccine against *S*. *japonicum* [7, 8]. Both native and recombinant purified Sjc26GST have been shown to provide a certain level of protection against infection, in terms of reduced worm burden, female fecundity, and egg viability [9–12]. We have also reported that reSjc26GST can be used for diagnosis of schistosomiasis in buffaloes, and that it provides high sensitivity and specificity [13]. In recent years, Sjc26GST has been developed into a DNA vaccine with the capacity to potentiate mainly Th1 immune responses against *S*. *japonicum* [14–16]. However, the effectiveness of the Sjc26GST DNA vaccine in reducing the worm burden was not significantly elevated, although we previously demonstrated that T helper type 1 (Th1) responses are important in providing protective immunity against schistosome infection [17].

The effectiveness of DNA vaccination alone is limited, because it often generates only a weak cellular immune response; therefore, the complementary use of adjuvants may be required to improve vaccine potency and enhance its immunoprotective effects against *S*. *japonicum* [15, 18, 19]. IL-12, which is involved in the differentiation of naïve T cells toward Th1 [20], is an effective adjuvant in increasing the protective immunity from vaccination with rSm14 against *S*. *mansoni* [21], as well as with Sj23 plasmid DNA against *S*. *japonicum* [22]. IL-12 co-administration with DNA vaccine priming can induce strong cell-mediated type 1 immune responses [20, 23]. Although Th1 immune responses are important in providing protective immunity against schistosome infection [21, 24, 25], a rapidly induced and excessive Th1 response may also cause damage to tissues of the infected host during parasite killing [26]. In addition, it has been shown that different adjuvants may be appropriate for various purposes, including prolonged antigen release, activation of nonspecific immune stimuli, and even reduction of side effects [27]. Research with a novel finding has shown that an immunization strategy employing combined DNA and recombinant protein vaccines can induce strong cellular and humoral responses [28]. Recently, this immunization strategy has also been used to provide a basis for optimizing vaccination against schistosomiasis japonicum [29–31].

In this study, we used pIL-12 as an adjuvant and co-immunized with recombinant SjGST (rSjGST) in an attempt to improve the protective efficacy of the SjGST DNA vaccine against *Schistosoma japonicum*. We found that combining pcDNA/SjGST with pIL-12 and rSjGST for immunization of mice increased the protective efficacy and simultaneously reduced pathologic injury of the liver caused by granulomas.

## Materials and Methods

### DNA plasmid construction

**Fig 1:**
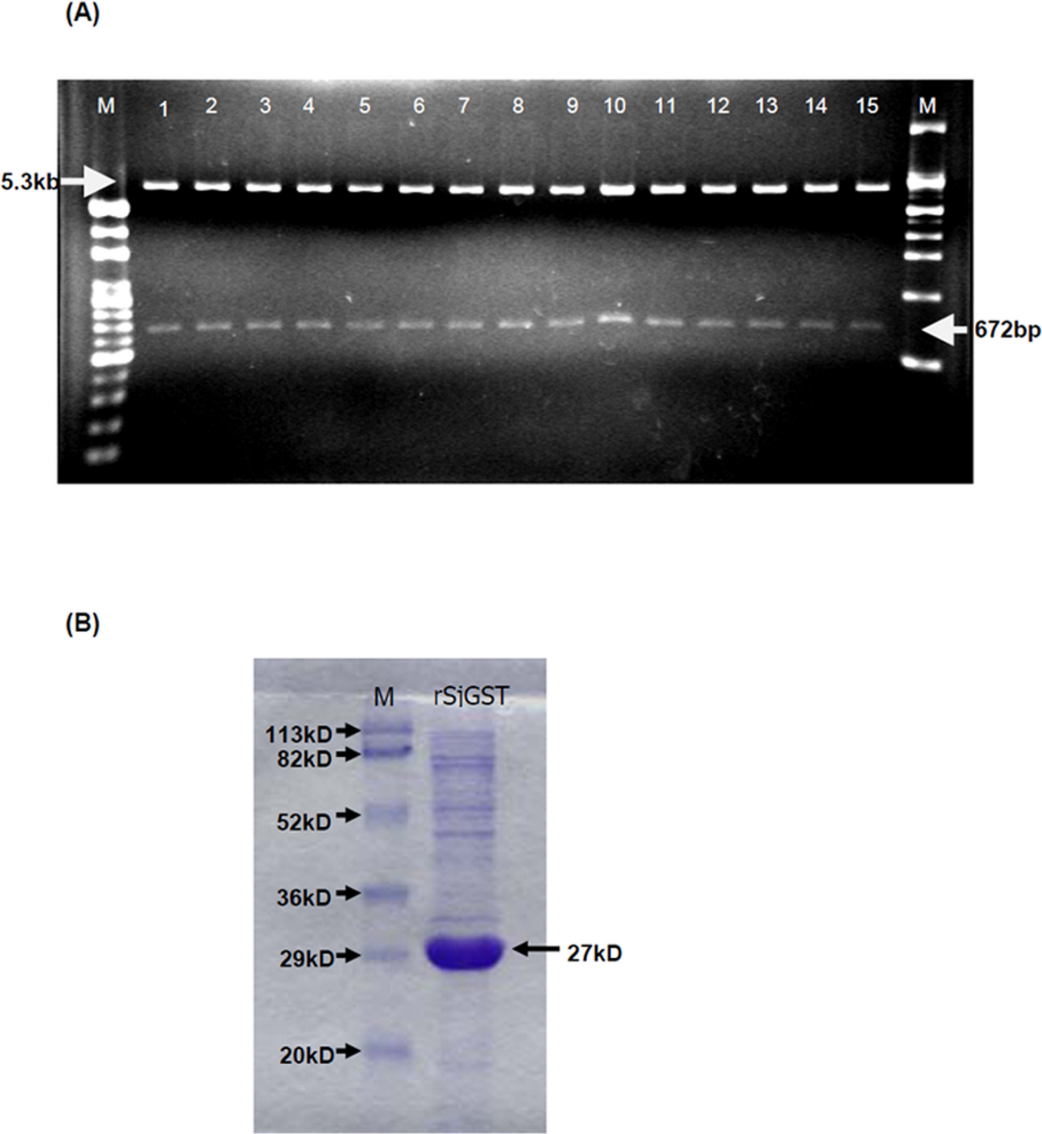
Confirmation of pcDNA/SjGST plasmid constructs and rSjGS protein. The pcDNA/SjGST plasmid and purified rSjGST were confirmed by agarose gel electrophoresis and SDS-PAGE, respectively. (A) The restriction enzyme EcoRV/HindIII digestion products for the SjGST gene are 672 bp. Lanes 1 to 15 showed 15 plasmids extracted from individual clones of *E*. *coli*. The sizes of all nucleotide sequences were as expected. (B) *E*. *coli*-derived rSjGST was extracted and purified by a Ni-NTA affinity column, and the purity of rSjGST was determined by SDS-PAGE.

The DNA vaccine construct was modified from pQE31/Sjc26GST, which contains the intact open reading frame (ORF) of 26 kDa SjGST from *Schistosoma japonicum* (GenBank accession no. BU711548.1) [13]. Briefly, the intact ORF of Sjc26GST was amplified by PCR using the pQE31/Sjc26GST vector as a template and primers containing EcoRV and BamHI restriction sites (Forward-5′-CGGGATCCCGTCATGTCCCCTATACTAGGTTAT-3′ and Reverse-5′-GGGATATCCCTTTATTTTGGAGGATGGTCGCCA-3′). The gene products was digested with *Bam*HI/*Eco*RV at 37°C for 4 h, and then subcloned into the pcDNA4 vector (Invitrogen, Waltham, MA, USA) at 37°C overnight to generate the pcDNA/SjGST construct. Constructs were confirmed by restriction analysis and sequencing (Fig 1A). The DH5α strain of *Escherichia coli* (Invitrogen, USA) was transformed with plasmid DNA. The DNA vaccine was amplified and purified according to the manufacturer’s instruction for endotoxin-free Giga Prep Kits (Qiagen, Valencia, CA, USA). The plasmid DNA vaccine was resuspended in 5% glucose and stored at −80°C until use. In addition to pcDNA/SjGST, we used rSjGST, prepared previously (Fig 1B) [13], and an IL-12-encoding plasmid (pIL-12), kindly provided by the laboratory of Dr. Tauo [32], as an adjuvant to enhance the immune response.

### Animals, vaccination, and infection

**Fig 2:**
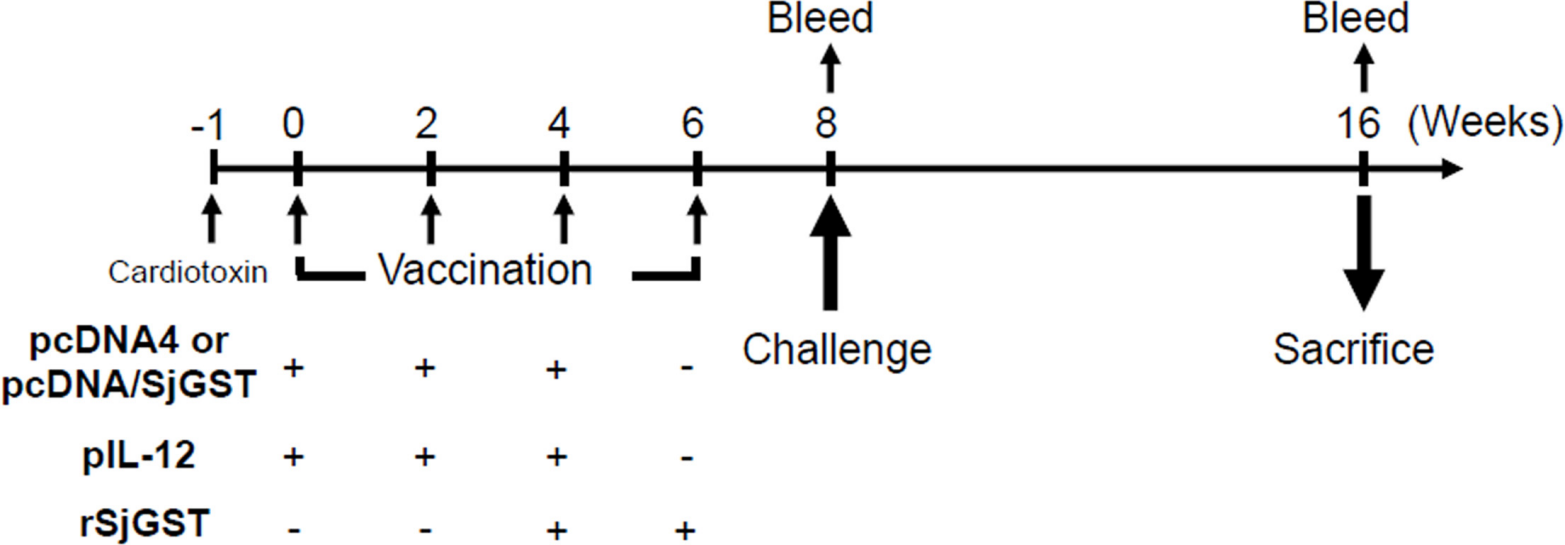
Time-schedule of animal vaccination, bleeding, infection, and sacrifice. The prime and boost immunization strategies for different groups were as shown. Briefly, Groups II, III, and IV were boosted in weeks 2 and 4; Group V was boosted in weeks 4 and 6; Group VI was boosted in weeks 2, 4, and 6. In week 6, Groups V and VI were only boosted with rSjGST. Eight weeks after vaccination with pcDNA/SjGST vaccine, pIL-12 adjuvant, or recombinant SjGST protein, the mice were challenged with 30 cercariae of *Schistosoma japonicum*. Mice were bled and sacrificed in 8 week after schistosoma infection. The total number of experimental animals in each groups ranged from 19–21, except in Group I (n = 8).

The mouse immunization protocol is shown in Fig 2. One week before vaccination, all C57BL/6 mice (6-week-old males) were intramuscularly primed with 100 μl (10 μM) of cardiotoxin in the quadriceps femoris muscle to enhance the permeability of the cellular membrane prior to the first DNA vaccination [33]. Mice were divided into six groups, each of which included 6–8 mice. The mice in Group I (untreated infection control) were pretreated with cardiotoxin only; mice in Groups II and III were immunized with 100 μg of control plasmid (pcDNA4) or 100 μg of pcDNA/SjGST alone, respectively; mice in Group IV were co-immunized with 100 μg of pcDNA/SjGST + 100 μg of pIL-12; mice in Group V were co-immunized with 100 μg of pcDNA/SjGST + 100 μg of rSjGST; mice in Group VI were immunized with a combination of 100 μg of pcDNA/SjGST, 100 μg of pIL-12, and 100 μg of rSjGST. Mice were boosted two or three times at 2-week intervals (weeks 2, 4, 6) following the initial vaccination in a manner specific for each group. After the final immunization, each group of mice was challenged with 30 cercariae by direct skin penetration through the abdomen at week 8, as previously described [34]. All experiments were performed in triplicate to ensure the reliability of the data.

### Analysis of anti-SjGST antibodies *in sera*

Blood was collected from the tail vein of the mice at week 8 (after vaccination) and week 16 (after infection). ELISA was used to determine the anti-SjGST antibody titer. In brief, 96-well plates were pre-coated with purified rSjGST (5μg/well) at 4°C overnight. FBS (5%) was added into each well to block non-specific binding and incubated at 37°C for 2 h. Diluted serum (1:20 dilution, 200μl) was added and incubated at 37°C for 2 h. HRP-conjugated goat anti-mouse IgG, IgG1, IgG2a, or IgG2b (1:2000 dilution) was added and incubated at 37°C for 1 h. ABTS (Zymed) was used as a substrate, and the optical density was measured at 405 nm using an ELISA reader (Dynatech MR5000).

### Assessment of worm burdens and hepatic egg burdens

After 8 weeks of infection, mice were bled, sacrificed, and perfused. Worms were collected from the hepatic portal system and counted by anatomic microscopy. Part of the mouse liver was cut, weighed, and digested with 5 ml of 5% KOH at 37°C overnight, then the egg number per gram was determined under microscope and calculated. The reduction rate was calculated using the following formula: Worm burden reduction rate (%) = (1—mean number of worms in immunized mice/mean number of worms in mice immunized with the control plasmid) × 100. Egg reduction rate (%) = (1—mean number of eggs per gram in immunized mice/mean number of eggs per gram in mice immunized with the control plasmid) × 100.

### Isolation of spleen immune cells

The method was modified as our previously reports described [35]. The spleens of sacrificed mice were removed and placed in a Petri dish and then washed to flush out the spleen immune cells. The spleen immune cells were dispersed using a PBS-filled syringe equipped with a 23-G needle. Residual red blood cells were lysed in phosphate buffered solution (PBS) containing 150 mM ammonium chloride, 1 mM potassium bicarbonate, and 0.1 mM ethylenediaminetetraacetic acid (EDTA). The intact spleen immune cells were recovered by centrifugation (500 ×g, for 3 min, 4°C), and resuspended for later use. Routinely, 95% of the isolated cells were viable, as determined by Trypan Blue exclusion assay.

### Real-time quantitative reverse transcription-polymerase chain reaction

mRNA quantification was assessed by real-time polymerase chain reaction (PCR). Total RNA was extracted using TRIzol™ reagent (Life Technologies, Carlsbad, CA, USA) and reverse transcribed with Moloney Murine Leukemia Virus (MMLV) reverse transcriptase (Promega, Madison, WI, USA) to generate cDNA. Each cDNA pool was stored at -20°C until further real-time PCR analysis. Primer pair specificity was validated by performing a RT-PCR reaction using common reference RNA (Stratagene, La Jolla, CA, USA) as a DNA template. The primers used are: Interleukin (IL)-2, forward: 5'-ATGGACCTACAGGAGCTCCTG-3', reverse: 5'-TCAAATCCAGAACATGCCGCAG-3'; IL-12, forward: 5'-ACATGGTGAAGACGGCCAG-3', reverse: 5'-GAAGTCTCTCTAGTAGCCAG-3'; Interferon-gamma (INF-γ), forward: 5'-CTTCCTCATGGCTGTTTCTG-3', reverse: 5'-TGTCACCATCCTTTTGCCAG-3'; IL-4, forward: 5'-CAGAGAGTGAGCTCGTCTG-3', reverse: 5'-GGTGCAGCTTATCGATGAATC-3'; IL-10, forward: 5'-ATGCAGGACTTTAAGGGTTAC-3', reverse: 5'-CCTGAGGGTCTTCAGCTTC-3'; Tumor Necrosis Factor-alpha (TNF-α), forward: 5'-CCTCACACTCAGATCATCTTC-3', reverse: 5'-CGGCTGGCACCACTAGTTG-3'; Glyceraldehyde-3-phosphate dehydrogenase (GAPDH) and 18s were used as endogenous reference genes. 18s, forward: 5'-AAGACGGACCAGAGCGAAAGCA-3', reverse: 5'-ATCGCCAGTCGGCATCGTTTATG-3'; GAPDH, forward: 5'-TCCTGGTATGACAATGAATACGG-3', reverse: 5'-GATGGAAATTGTGAGGGAGATG-3'. Real-time PCR reactions were performed on the lightcycler nano real-time PCR system (Roche Diagnostics, Mannheim, Germany) using LightCycler 480 SYBR Green I Master (Roche Diagnostics). Briefly, 10 μl reactions containing 2 μl of Master Mix, 2 μl of 0.75 μM forward primer and reversed primer, and 6 μl of the cDNA sample. Each sample was run in triplicate. The real time PCR program were for 3 minutes at 95°C; 45 cycles for 10 seconds at 95°C, 30 seconds at 60°C. At the end of the program, a melt curve analysis was done. Data analysis was performed using the LightCycler Nano software version 1.0 (Roche).

### Histopathological examination of liver tissue for granulomas

The entire livers of sacrificed mice were collected, fixed, and then stained with hematoxylin and eosin, as described previously [36]. The extent of pathological changes in the liver was assessed macroscopically on the day of sacrifice. Briefly, liver specimens were fixed in 10% formalin, embedded in paraffin, and sectioned at 3-μm thickness. The sizes of non-confluent granulomas formed around a single egg were measured in the stained sections using the ImageJ software (NIH). For all histological quantification, 7 mice livers from each group were analyzed and at least 3 slides were used to determine the width of the granuloma borders of each specimen by referring the previous description [37]. Two liver sections, selected to be sufficiently distant from each other to ensure that a granuloma was not measured twice, were used for all granuloma measurements.

### Statistical analysis

Data were collected from three independent experiments. Antibody and cytokine levels were compared by one-way ANOVA using SPSS 18.0 software (SPSS Inc. Chicago, IL, USA). Other results were analyzed using two-tailed Student’s *t*-test. In all analyses, differences of *p* < 0.05 were considered significant.

## Results

### Specific antibody responses of mice after immunization and after infection with *S*. *japonicum*

**Fig 3:**
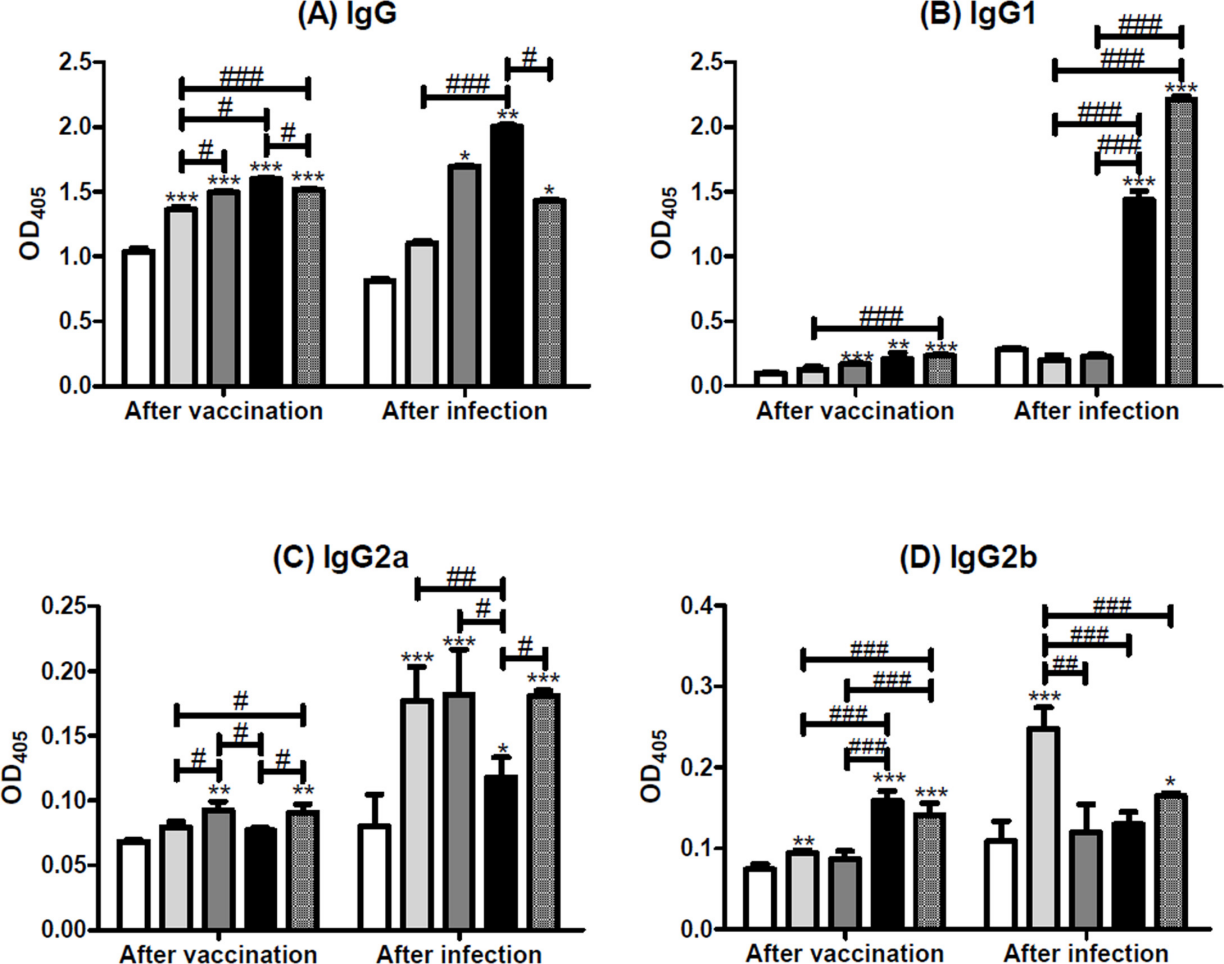
The level of anti-SjGST in serum samples after vaccination and after *Schistosoma japonicum* challenge. Serum samples were collected from the tails of mice immunized with pcDNA4 (white), pcDNA/SjGST (light grey), pcDNA/SjGST + pIL-12 (dark grey), pcDNA/SjGST + rSjGST (black), or pcDNA/SjGST + pIL-12 + rSjGST (mosaic), 8 weeks after vaccination (left panel) or 8 weeks after infection (right panel). The antibody titers of (A) total IgG, (B) IgG1, (C) IgG2a, and (D) IgG2b were measured by ELISA. The OD405 value of each group was directly shown in the figure. The results are shown as the mean ± standard deviation from three independent experiments. *: *p* < 0.05; **: *p* < 0.01; ***: *p* < 0.001, compared with the control group treated with pcDNA4. #: *p* < 0.05; ##: *p* < 0.01; ###: *p* < 0.001, compared between the two indicated groups.

Eight weeks after vaccination with pcDNA/SjGST alone (Fig 3A left bars), the titer of anti-rSjGST IgG increased more than pcDNA4 control group. Levels of anti-rSjGST IgG antibodies in mice vaccinated with pcDNA/SjGST + pIL-12 alone (Group IV), + rSjGST alone (Group V), or + pIL-12 and rSjGST (Group VI) were significantly higher than those in Group II or III (p <0.001; p <0.05). Furthermore, the dominant antibody isotypes in mice vaccinated with pcDNA/SjGST plus rSjGST were IgG1 and IgG2b (p <0.01; p <0.001) (Fig 3B and 3D, left bars). Immunization with a combination of pcDNA/SjGST and pIL-12 significantly induced IgG2a production (p <0.05) (Fig 3C, left bars).

On the other hand, eight weeks after infection with *S*. *japonicum*, sera were collected and the mice were sacrificed. We found that levels of anti-SjGST specific total IgG were higher in the infected mice vaccinated with pcDNA/SjGST + pIL-12, pcDNA/SjGST + rSjGST, or plus both pIL-12 and rSjGST (Group IV–VI) than in the control group mice vaccinated with pcDNA4 (Group II) (p <0.05) (Fig 3A, right bars). However, Fig 3B (right bars) showed that IgG1 was strongly elicited only in mice vaccinated with pcDNA/SjGST + rSjGST, whether pIL-12 was included or not (Group V–VI) (p < 0.001); vaccination with pcDNA/SjGST sufficiently induced the production of IgG2a except in Group V that plus rSjGST alone (p < 0.001) (Fig 3C, right bars); and only vaccination with pcDNA/SjGST alone (Group III) induced specific IgG2b (p < 0.001) (Fig 3D, right bars).

### Protective effect of DNA vaccine

**Fig 4:**
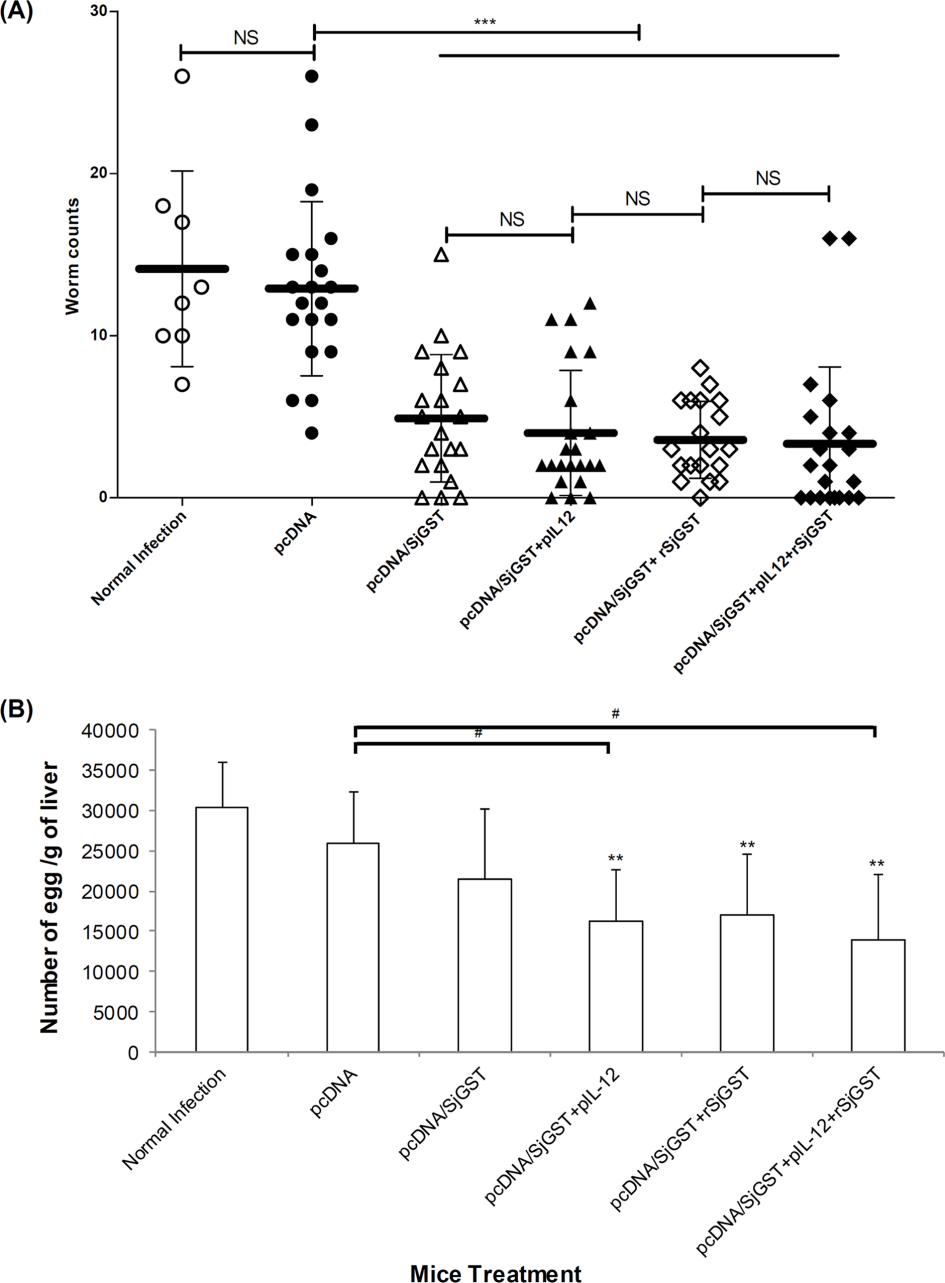
The worm and hepatic egg burdens of vaccinated animals challenged with *Schistosoma japonicum*. Six groups of mice were immunized with pcDNA4 vehicle, pcDNA/SjGST vaccine, pIL-12 and rSjGST. The vaccinated mice were infected with 30 cercaria/ mouse. Each group included 19–21 animals (with the exception of Group I, n = 8) from three independent experiments. Eight weeks after schistosome infection, (A) adult worms were collected from infected mice and counted under an anatomy microscope. Each point represents the number of worms in a single mouse. The horizontal black lane represents the mean ± SD of that group. NS: not significant; ***: p < 0.001 between different groups. (B) In addition, parts of the mice livers were collected, weighed, and digested for egg counting. Mean ± SD are shown. *: *p* < 0.05, **: *p* < 0.01, compared with the untreated infection control. #: *p* < 0.05, compared between the two indicated groups.

Vaccination with the pcDNA/SjGST plasmid efficiently induced production of specific anti-SjGST antibodies recognizing the rSjGST antigen. Next, we tested the protective efficacy against *S*. *japonicum* infection. The vaccinated mice groups were challenged with 30 cercariae. Eight weeks following infection, mice were bled and sacrificed. Adult worms were collected from the hepatic portal system after perfusion and the egg burden was measured. Fig 4A and Table 1 show the worm count for the control group (Group II, immunized with the pcDNA4 plasmid alone) did not differ from that in untreated infected mice (Group I). Compared to Group II, the worm burden of Group III was significantly reduced (the reduction rate was 62.02%) (p < 0.001). Co-immunization with pcDNA/SjGST + pIL-12 or rSjGST enhanced the worm reduction rate to 68.99% and 72.33%, respectively (p < 0.001). Combined vaccination with pcDNA/SjGST + pIL-12 + rSjGST sufficiently reduced the number of recovered worms, from 12.9 to 3.33 (reduction rate = 74.19%) (p < 0.001).

**Table 1:** The effect on worm and egg burdens in vaccinated mice.

Vaccination			Worm burden		Egg burden	
			Recovery (n ± SD)	Reduction (%)	Recovery (n ± SD)	Reduction (%)
Plasmid		Protein				
Immunogen	Adjuvant					
-	-	-	14.12 ± 6.03	-	30331.2 ± 5738.2	-
pcDNA4	-	-	12.90 ± 5.36	-	25867.9 ± 6460.4	-
pcDNA/SjGST	-	-	4.90 ± 3.93	62.02 ^###^	21450.6 ± 8788.9	17.08
pcDNA/SjGST	pIL-12	-	4.00 ± 3.85	68.99 ^###^	16155.8 ± 6554.2	37.54 ^#^
pcDNA/SjGST	-	rSjGST	3.57 ± 2.36	72.33 ^###^	16952.6 ± 7681.3	34.46
pcDNA/SjGST	pIL-12	rSjGST	3.33 ± 4.73	74.19 ^###^	13838.1 ± 8236.3	46.50 ^#^

# a significant reduction in worm and egg burdens recovered compared to mice immunized with pcDNA4 alone, using Student’s *t*-test.

**#:** *p* < 0.05

*###*: *p* < 0.001

The number of eggs in the liver tissue of each group is presented in Fig 4B and Table 1. Compared to Group II mice, the number of eggs per gram of liver in Group III was only reduced by 17.08%. However, the addition of pIL-12 to pcDNA/SjGST led to a significant reduction of the hepatic egg burden (37.54%, p < 0.05) compared to Group II mice. A significant difference (a decrease of 46.50%) was observed between the group co-immunized with pcDNA/SjGST + pIL-12 + rSjGST and the group immunized with pcDNA4 alone **(p < 0.05),** but not the group immunized with pcDNA/SjGST + rSjGST. Thus, vaccination of mice with pcDNA/SjGST + pIL-12 + rSjGST can significantly reduce the worm and hepatic egg burdens.

### Cytokine expressions of immunized mice after infection with *S*. *japonicum*

**Fig 5:**
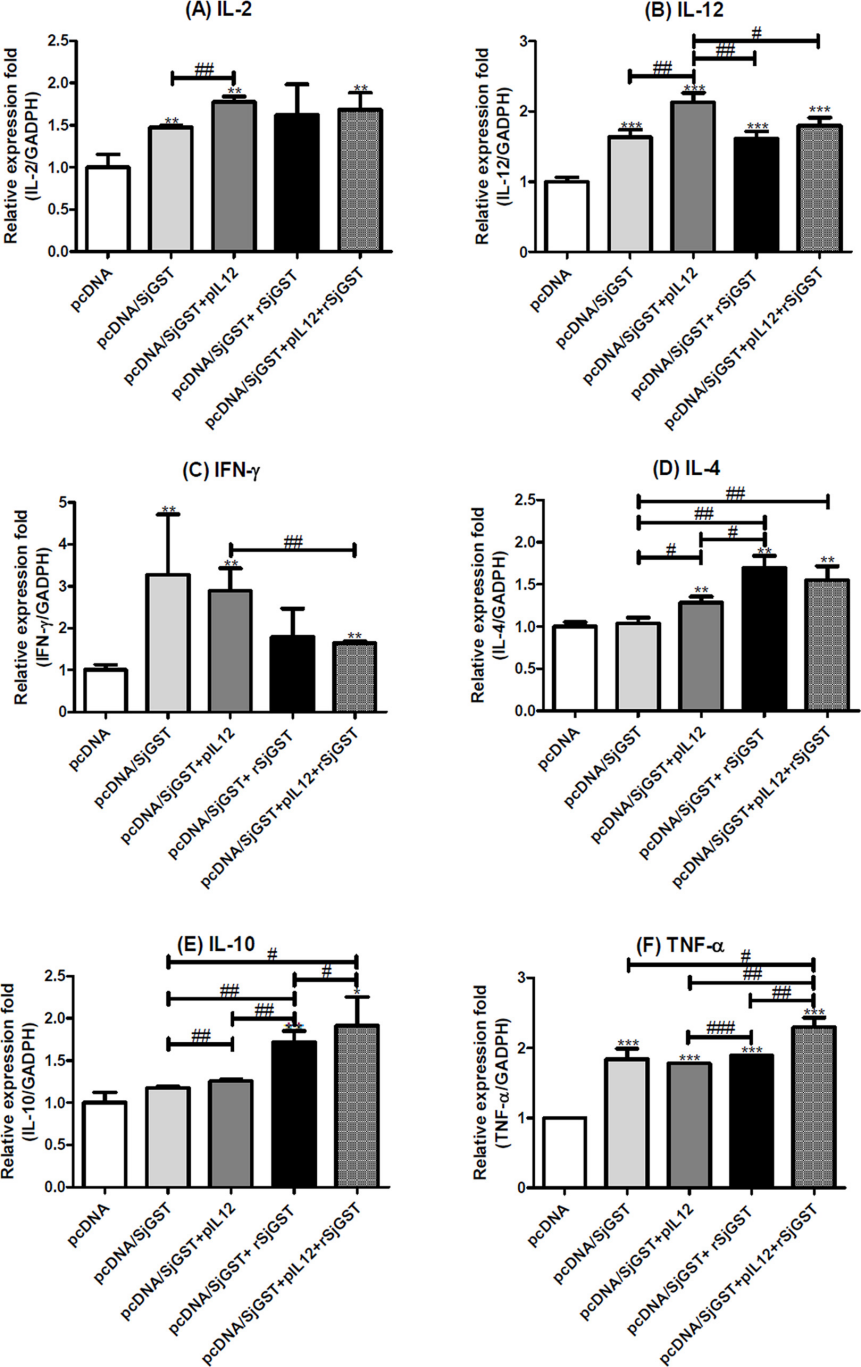
The cytokine mRNA expression levels in spleen cells after infection. Spleen immune cells were isolated from the spleen of mice 8 weeks after infection. The mRNA expression levels of (A) IL-2, (B) IL-12, (C) IFN-γ, (D) IL-4, (E) IL-10, and (F) TNF-α were measured by qRT-PCR. Comparative CT was used to determine the individual relative mRNA expression levels (fold differences) when normalized to the expression level of the internal control genes, GAPDH and 18s. The cytokine gene expression level of the pcDNA4 group was used as background to determine the relative expression. The results are shown as the mean ± standard deviation from three independent experiments. *: *p* < 0.05; **: *p* < 0.01; ***: *p* < 0.001, compared with the control group treated with pcDNA4. #: *p* < 0.05; ##: *p* < 0.01; ###: *p* < 0.001, compared between the two indicated groups.

Splenic immune cells were isolated immediately after mice were sacrificed and then subjected to gene expression assay. As shown in Fig 5A, mice vaccinated with pcDNA/SjGST showed significant induction of IL-2, except for Group V that plus rSjGST alone (p < 0.01) when compared with the pcDNA4 control. Mice vaccinated with pcDNA/SjGST, particularly when plus pIL-12 alone (Group IV), effectively upregulated IL-12 (p < 0.001) as well as INF-γ (p < 0.01) mRNA, with the exception of INF-γ expression in Group V (Fig 5B and 5C). Conversely, as presented in Fig 5D and 5E, the Th2 IL-4 gene and immunosuppressive IL-10 gene were significantly upregulated when rSjGST was used as an adjuvant whether pIL-12 was included or not (p < 0.01; p < 0.05). IL-4 mRNA levels in Group V were much higher than in the other groups without rSjGST (p < 0.05), whereas IL-10 expression was the highest in the group vaccinated with the pcDNA/SjGST + pIL-12 + rSjGST combination (p < 0.05). It is noteworthy that TNF-α gene expression was significantly induced in all mice vaccinated with pcDNA/SjGST (p < 0.001). TNF-α mRNA expression was the highest in Group VI, compared to that in all of the other experimental groups (p < 0.05) (Fig 5F).

### Histopathological effect of the DNA vaccine

**Fig 6:**
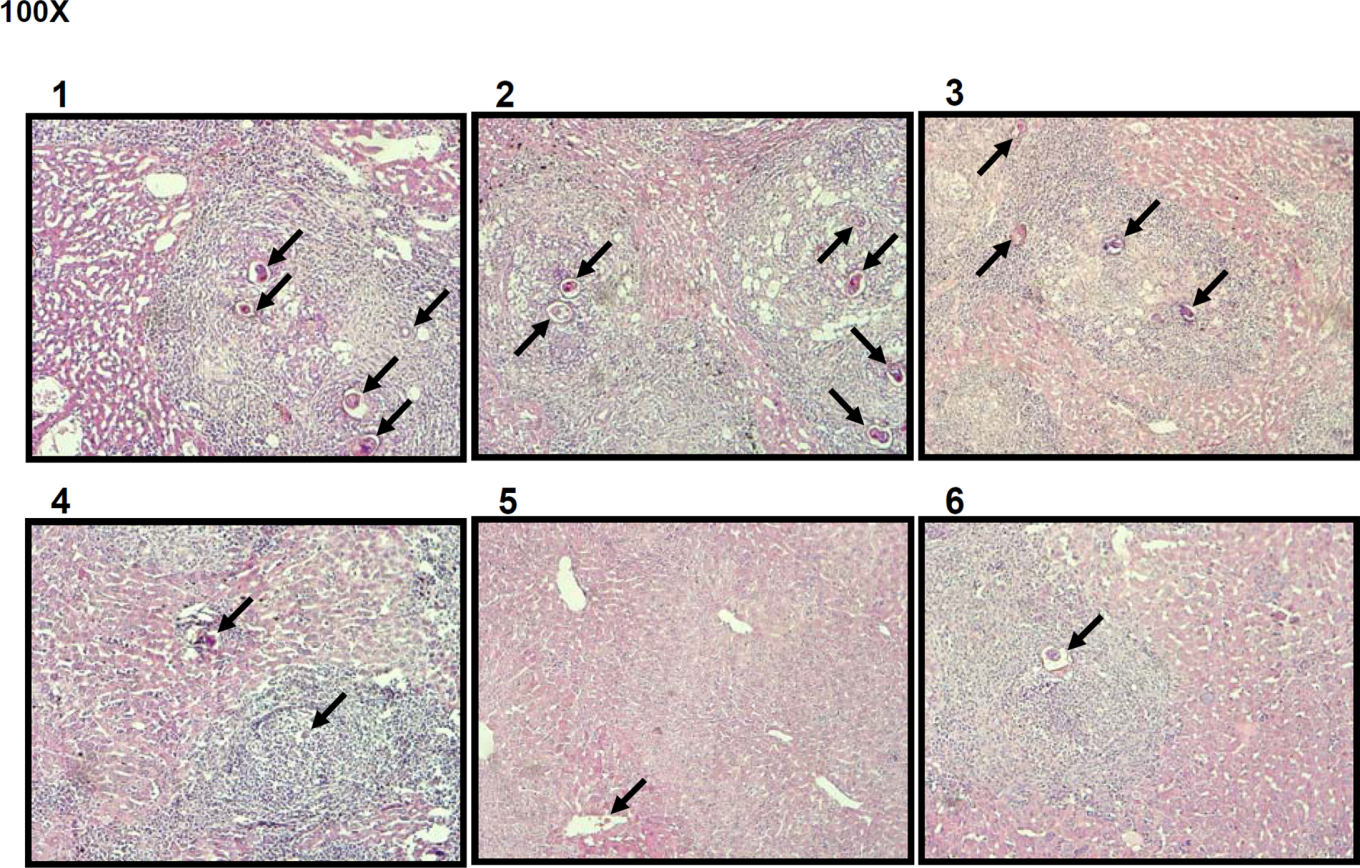
Histopathology of liver tissue sections of mice in different groups. Vaccination with a combination of pcDNA/SjGST vaccine, IL-12 plasmid, and recombinant SjGST protein reduced egg deposition and egg-induced granulomas. The numbers 1–6 above the images represent Group I-VI of mice, respectively. Group V and VI showed fewer inflammatory cells in the liver tissue. Paraffin-embedded liver tissues from *S*. *japonicum*-infected mice were stained using H&E. Tissue images were captured with a microscope (Leica) under 100 × magnifications. Sites of egg deposition are shown with arrows.

**Fig 7:**
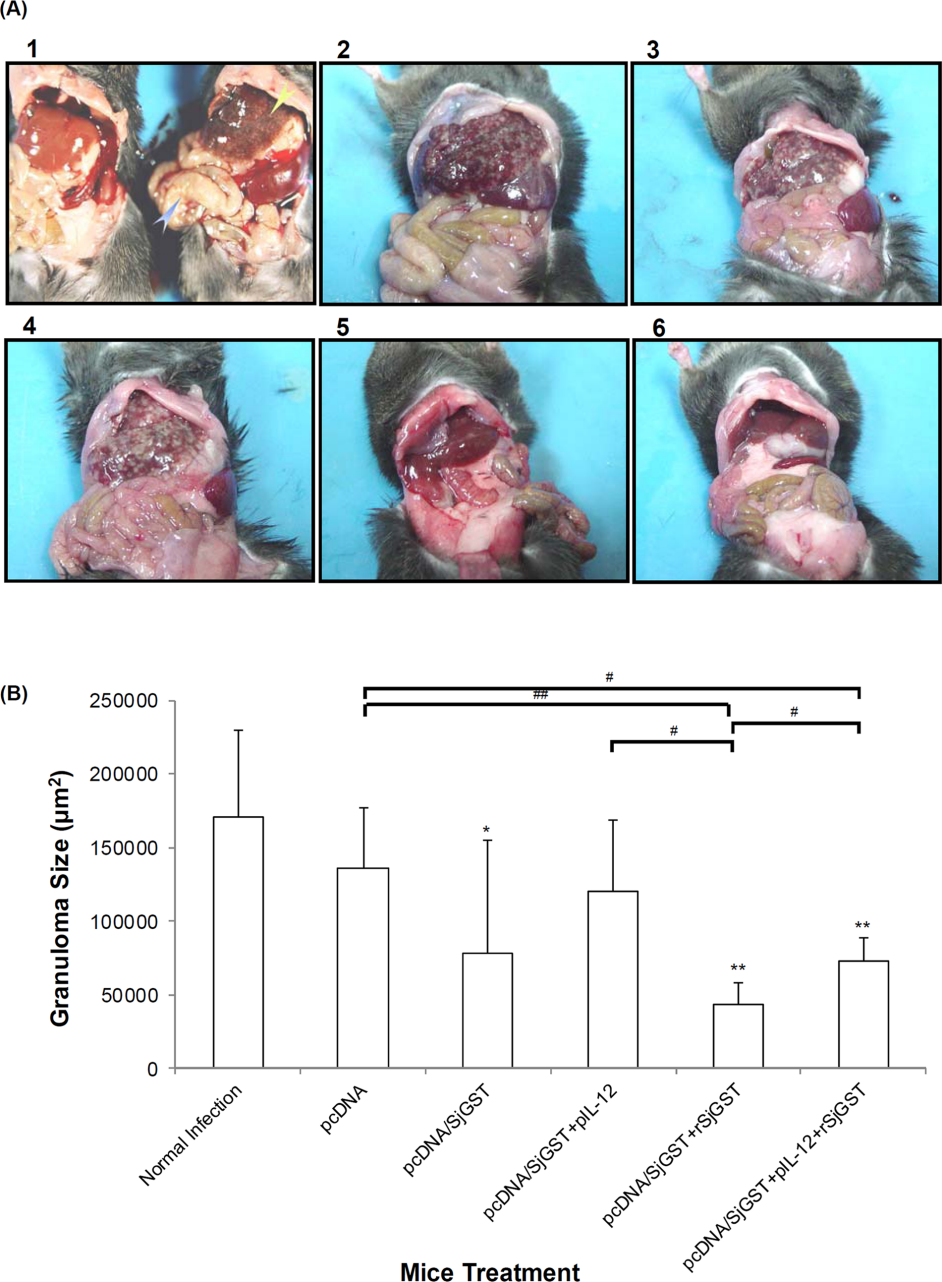
Hepatic morphology and average granuloma size in vaccinated animals challenged with *Schistosoma japonicum*. (A) Morphological differences were observed between livers of mice in different groups. Image 1 shows the liver and spleen of an uninfected (left) and infected (right) C57BL/6 mouse at 8 weeks. Images of Group V and VI show that vaccination with pcDNA/SjGST vaccine plus rSjGST reduced the pathological damage of egg-induced granulomas. (B) Size distribution of hepatic granulomas after vaccination with pcDNA/SjGST or addition of pIL-12 and rSjGST, followed by infection challenge. The size of each granuloma surrounding an egg was quantitated using ImageJ software (NIH). Results represent the average of 50 granulomas (mean ± SD, in μm^2^) from 7 animals per group. *: p < 0.05, **: p < 0.01, compared with untreated infected mice (Group I). #: p < 0.05, ##: p < 0.01 represent comparisons between the two groups indicated.

Liver damage caused by the egg-induced granulomas is the major pathogenicity of *S*. *japonicum* infection. This egg deposition results in the formation of granulomas. However, we found that liver pathology by granuloma formation was not entirely consistent with the reduction rates of egg deposition which significant reduced in Group IV and Group VI. As shown in Fig 6, deposition of many schistosome eggs and severe granuloma formation were observed in liver tissue collected from Groups I and II. After vaccination with pcDNA/SjGST or co-immunization with pIL-12, we found that granuloma pathogenesis was not significantly reduced (Fig 6, III–VI), while hepatic egg burdens were decreased (Table 1 and Fig 4B). However, few sites of egg deposition or granuloma were observed Groups V and VI, which were vaccinated with a combination of pcDNA/SjGST + rSjGST and pcDNA/SjGST + pIL-12 + rSjGST. Morphological observations of liver tissue from mice in these groups are concordant with these results (Fig 7A).

To determine whether granuloma formation would be affected in immunized mice, we measured the size of granulomas observed in the hepatic tissue samples. As shown in Fig 7B, mice immunized with pcDNA/SjGST exhibited smaller granulomas than mice in the untreated infection control group (p < 0.05). Moreover, the size of granulomas was not reduced in mice simultaneously co-immunized with pIL-12 as much as in other groups. In contrast, the area of egg-induced granulomas was significantly reduced in mice vaccinated with pcDNA/SjGST + rSjGST or pcDNA/SjGST + pIL-12 + rSjGST compared with mice vaccinated with pcDNA alone or untreated infected mice (p < 0.05; 0.01). In addition, Group V and Group VI were also different from each other (p < 0.05). This suggests that recombinant SjGST suppressed the excessive liver damage caused by hepatic granuloma. The above results show that vaccination with SjGST DNA and recombinant SjGST can protect mice from *S*. *japonicum* infection.

## Discussion

In the public health area, development of a vaccine is thought to be an efficient strategy to control schistosoma infection. The WHO recommends that the minimal protection level provided by candidate vaccines is 40% [38]. Several studies have shown that effective vaccination against schistosomes depends on the simultaneous induction of both cell-mediated and humoral immunity [39–42]. Using DNA priming/protein vaccine boosting protocols may facilitate achievement of this immunization goal. In this study, we focused on protection enhancement and pathogenesis reduction of the pcDNA/SjGST DNA vaccine, by co-immunizing with IL-12 plasmid DNA in addition to recombinant SjGST protein.

Immunization with SjGST DNA-based vaccines against the zoonotic pathogen *S*. *japonicum* has been much studied, even though they produce only a limited reduction in worm burden [14, 29, 43]. Insufficient uptake of intramuscularly delivered DNA plasmid leading to poor immune responses to antigens is one of the primary reasons underlying the overall low efficacy of DNA vaccines. Combining SjGST plasmid DNA vaccines with adjuvants, such as Cimetidine, IL-18, TLR ligands, levamisole, and others, could enhance the protection level [15, 18, 19, 44]. Here, we showed that our pcDNA/SjGST plasmid DNA provides efficient worm reduction (60%), up to >70% if used with the adjuvant pIL-12, rSjGST, or both. However, the anti-egg effects of vaccination with pcDNA/SjGST were not as good as the worm reduction effects, unless the mice were co-immunized with pIL-12, whereas the addition of both pIL-12 and rSjGST produced the highest egg reduction rate (46%). Use of IL-12 as a gene adjuvant induces production of IFN-γ and TNF-α cytokines, and enhances cytotoxic type-1 responses as well as protective immunity [21, 45]. Additionally, similar results were observed in our cytokine data (Fig 5). Moreover, vaccination experiments in pigs and buffalos have shown that combining IL-12 with a DNA vaccine can significantly improve the hepatic or fecal egg reduction rate and the female worm reduction rate [22, 46]. Cheng et al. confirmed the anti-fertility effect of IL-12, which can lead to impaired worm development and a significantly decreased number of eggs per pair of worms in the liver as well as in the uteri of ovigerous females [47].

Siddiqui et al. found that pIL-12 co-injected with Sm-p80 DNA yielded an augmentation of total IgG and IgG2a, predominantly of Th-1–type antibodies [23, 48]. Our data showed that vaccination with pcDNA/SjGST alone or a combination of pcDNA/SjGST and pIL-12 induce sufficient levels of total IgG and Th1-associated IgG2a to protect mice from schistosome infection. Furthermore, IgG2a and IgG1, Th1- and Th2-associated antibodies, were the dominant subtype detected in mice co-immunized with pIL-12, rSjGST, and pcDNA/SjGST. In addition, the gene expression data demonstrated that immunization with pcDNA/SjGST, particularly with pIL-12 as an adjuvant, induced significant Th1-associated IL-12 and INF-γ cytokines. When rSjGST was included in the immunization program, the expression of Th2 and Treg cytokines, IL-4 and IL-10, was increased markedly without leading to a decline in IL-12 expression levels. Our data show that co-vaccination with pcDNA/SjGST vaccine plus pIL-12 and rSjGST can induce parasite-specific humoral immunity but does not interfere with the protective efficacy of cell-mediated immune responses. Excessive Th1 cytokines that rapidly expand in the acute stage of infection may cause lethal immunopathology in a parasitized host [26, 49], whereas suitable Th2 cytokines can attenuate excessive inflammatory injury and promote tissue repair [50, 51].

Although previous reports have shown that co-immunization with a recombinant vaccine and rIL-12 can reduce the size of hepatic granulomas in infected mice [21], and even IL-12 itself can reduce the size of granulomas [47, 52], other studies have indicated that vaccination with a plasmid DNA vaccine plus an IL-12-expressing plasmid might instead increase hepatic damage [22]. Recombinant IL-12 and plasmid IL-12 DNA as adjuvants co-immunized with a DNA vaccine may have a different effect, because the plasmid DNA adjuvants might amplify and sustain the immune effect of IL-12 by cell expression [53]. DNA vaccines themselves also induce Th1-type cellular responses with high levels of IFN-γ production [43]. Therefore, co-immunization of pcDNA/SjGST with pIL-12 may induce an excessive Th1 response in a short time. Nonetheless, IL-12 was still indispensable in our vaccination regimen, improving protective immunity and anti-egg effects of the pcDNA/SjGST vaccine.

Using a recombinant protein vaccine to boost the effect of a DNA vaccine has been advanced as a new regimen for development of vaccines against schistosomiasis [31], even in the application of SjGST vaccine [29, 30]. Our results show that pcDNA/SjGST vaccination by boosting with recombinant SjGST proteins not only enhanced the anti-parasite efficacy against schistosomiasis (Table 1 and Fig 4A) but also significantly increased the anti-pathological effects, as evident in the reduction in the quantity and size of liver granulomas observed in the rSjGST-boosted groups (Fig 4B and Fig 7B). The positive impact of the SjGST boost on induced Th2 immunity was also demonstrated by inducing SjGST-specific IgG1 antibody and Th2 cytokine increases; in particular, IgG1 and IgG2a levels as well as Th1 and Th2 cytokines were elevated with pIL-12 and rSjGST co-immunization. The Th2 cytokines, IL-4 and IL-13, can suppress excessive neutrophil recruitment and proinflammatory cytokine production, which are mainly responsible for hepatic damage during schistosomiasis japonica infection [54]. Thus, rSjGST boosting may play a role in alleviating hepatic damage caused by strong Th1-promoting DNA vaccines, plus adjuvants such as pIL-12.

Results of cytokine expression explained the possible mechanism of additive effects as co-immunization with pcDNA/SjGST + pIL-12 and rSjGST. Immunization of pcDNA/SjGST alone or pcDNA/SjGST + pIL-12 both rapidly enhanced Th1 responses, e.g. IL-2, IL-12 and INF-γ that are important in providing protective immunity against *S*. *japonicum* [48, 55]. This effect is particularly significant when pcDNA/SjGST combining with pIL-12 and no matter plus rSjGST or not. During schistosoma infection, TNF-α participated not only the cell-mediated protective immunity, but also involved in the immune responses that affect liver fibrosis and hepatosplenomegaly [56, 57]. Our data showed that the pcDNA/SjGST vaccine induced significant levels of TNF-α and was the highest in Group VI with both pIL-12 and rSjGST as adjuvants. Meanwhile, rSjGST protein added to the immunization protocol induced the expression of Th2 cytokines IL-4, and especially IL-10. IL-10 plays a crucial role in regulating not only the severity of acute liver pathology, but also granulomatous organization and cohesiveness [58, 59]. Egg-induced inflammation, large granulomas, hepatic fibrosis, and tissue eosinophilia were observed in IL-10/IL-12-deficient mice with schistosome infection [49]. In the acute stage of schistosome infection, tolerance to worm egg antigens led to the enhancement of the Th1 response and a reduction in the Th2 response, as well as an increase in host mortality rate [60]. Therefore, inducing the appropriate Th2 response will decrease unnecessary damage to host tissue within immune attacks.

We have developed a new vaccination regimen against schistosomiasis japonica. Co-immunization of mice with pIL-12, rSjGST, and a pcDNA/SjGST vaccine produced significant anti-parasite, anti-hepatic egg, and anti-pathology effects. This regimen can induce both specific cellular and humoral responses to attain a balance between parasite elimination and prevention of pathological tissue injury. The efficacy of our method of vaccination should be further validated in large animals, such as water buffalo. These results may help to reduce the transmission of zoonotic schistosomiasis japonica.
